# Inflammatory myofibroblastic tumor arising from the ascending aorta mimicking a thymoma

**DOI:** 10.1007/s11748-019-01231-2

**Published:** 2019-10-22

**Authors:** Ju Sik Yun, Sang Yun Song, Kook Joo Na, Seok Kim, Yoo Duk Choi

**Affiliations:** 1 Department of Thoracic and Cardiovascular Surgery, Chonnam National University Hwasun Hospital, Chonnam National University School of Medicine, 322 Seoyang-ro, Hwasun-eup, Hwasun-gun, Jeollanam-do, South Korea; 2 Department of Pathology, Chonnam National University Hospital, Chonnam National University School of Medicine, Gwangju, South Korea

**Keywords:** Inflammatory myofibroblastic tumor, Aortic neoplasm, Thymoma

## Abstract

An inflammatory myofibroblastic tumor originating from the aorta is extremely rare. Here, we report a case involving a 41-year-old female patient with an aortic inflammatory myofibroblastic tumor. Although preoperative imaging showed a mediastinal mass indicative of invasive thymoma, surgical findings revealed that this mass originated from the aorta. The patient underwent partial resection of the aorta, including the mass with patch angioplasty. Based on the postoperative histological findings, the patient was diagnosed with an aortic inflammatory myofibroblastic tumor and is currently under outpatient follow-up.

## Introduction

Inflammatory myofibroblastic tumor (IMT) is a rare neoplasm consisting of myofibroblastic spindle cells and various inflammatory cells. Although these tumors are usually considered benign, local recurrence and distant metastasis are commonly observed, suggesting the intermediate biological potential of these tumors. Since the first report of a pulmonary IMT, these tumors have been reported mostly in the lungs; however, extrapulmonary lesions, such as in the abdomen, retroperitoneal space, pelvis, and extremities, have also been reported [1].

## Case

**Fig. 1:**
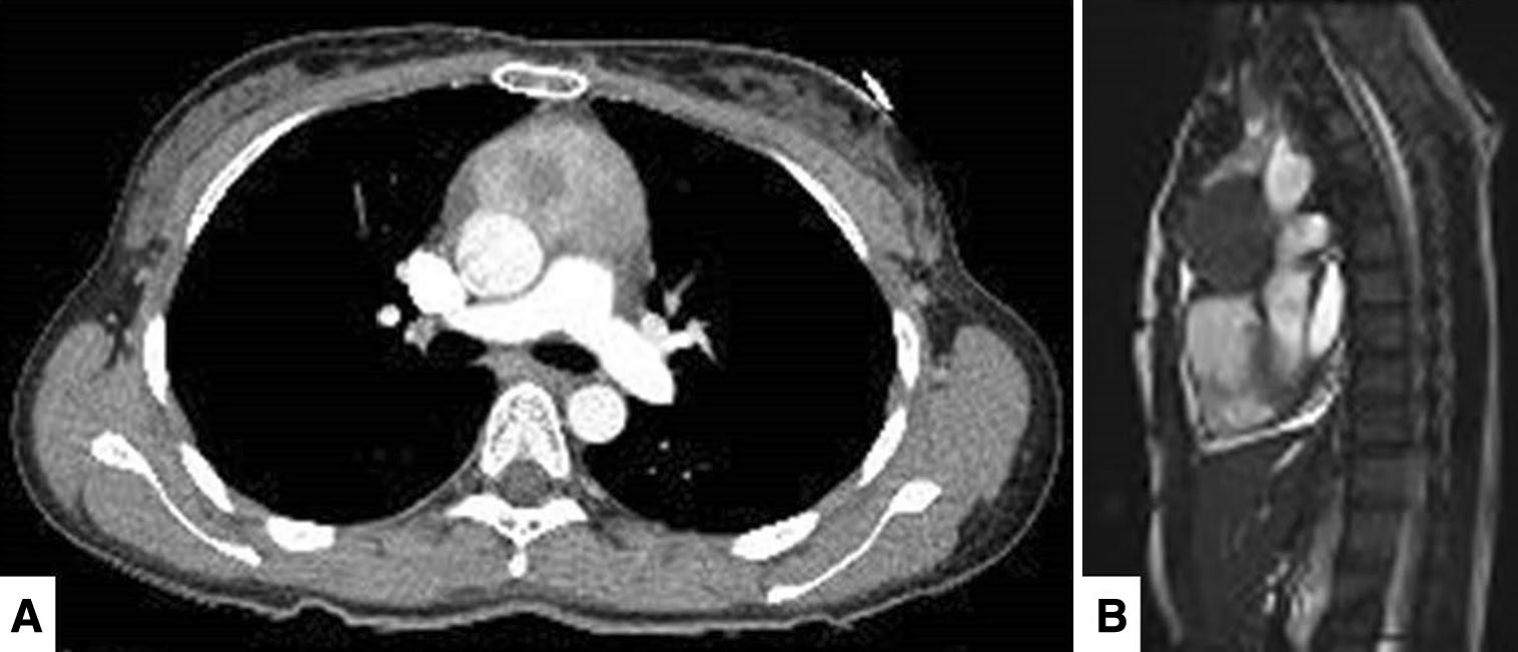
**a** Chest CT scan (axial section) shows a relatively well-defined heterogeneously enhanced mass in the anterior mediastinum. **b** Cardiac MRI (sagittal section) shows a mass compressing the ascending aorta and proximal main pulmonary artery. *CT* computed tomography, *MRI* magnetic resonance imaging

A 41-year-old female patient was admitted to the department of cardiology at our center, with the chief complaint of chest discomfort. Echocardiography revealed moderate pericardial effusion. Chest computed tomography (CT) revealed a 5.5-cm mass in the anterior mediastinum, along with pericardial effusion (Fig. 1a). The patient’s medical history was unremarkable, and routine preoperative examination findings were normal, except for slight elevation of the white blood cell count (11,900 cells/mm^3^). Cardiac magnetic resonance imaging (MRI) was performed, and while the boundaries of the mass were relatively clear and distinct, the possibility of its invasion into the aorta and pulmonary artery could not be completely ruled out (Fig. 1b).

Based on the preoperative imaging results, we suspected the patient of having invasive thymoma and planned to resect the mass by median sternotomy. However, contrary to our expectation, the mass was confirmed to be located in the intrapericardial space and not in the anterior mediastinal space. Following partial pericardiotomy, we could identify an irregular and solid mass in the upper region of the ascending aorta and proximal main pulmonary artery. The mass was carefully dissected from the right ventricular outflow tract, and we confirmed that it originated in the anterior wall of the aorta. We performed cardiopulmonary bypass through the femoral artery and right atrium to achieve complete resection. With sufficient safety margin (≥ 1 cm), partial resection of the aorta including the remnant mass and Dacron patch angioplasty was successfully performed. Total cardiopulmonary bypass time was 45 min (aortic cross-clamp time = 20 min).

**Fig. 2:**
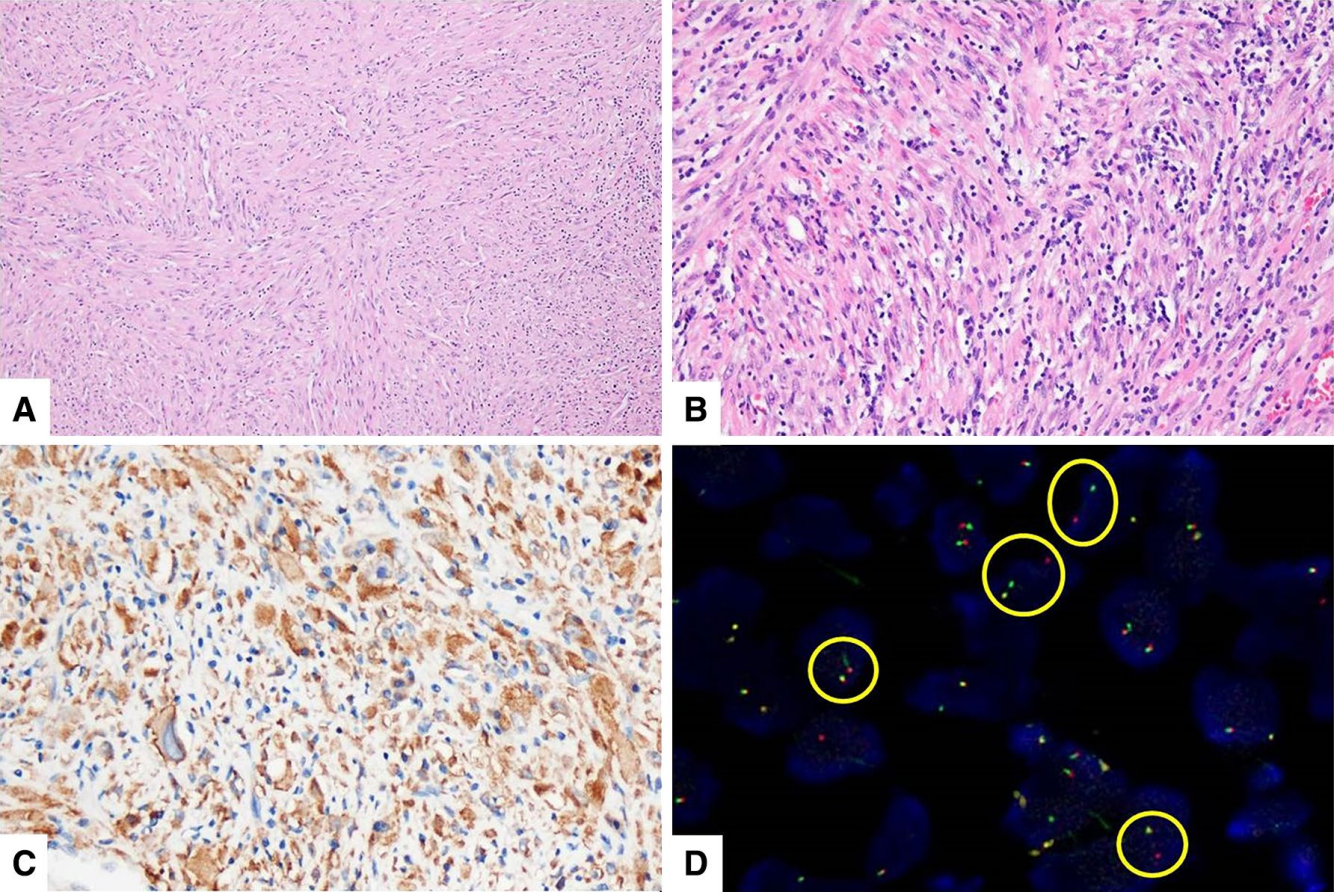
Histopathologic findings (hematoxylin and eosin staining) show **a** a proliferation of bland myofibroblastic spindle cells within the loose fibrous background and **b** the spindle cells containing small nuclei with vesicular chromatin mixed with inflammatory cells (mainly lymphocytes and plasma cells with occasionally eosinophils and neutrophils). Mild cytologic atypia and pleomorphism are observed. **c** Immunohistochemical staining shows that the spindle tumor cells are positive for ALK and **d** ALK break-apart FISH positive specimen shows splitting red and green signals (circles). *ALK* anaplastic lymphoma receptor tyrosine kinase, *FISH* fluorescence in situ hybridization

The patient was discharged on postoperative day 8, without any complications. Final pathology revealed that the tumor was composed of bland-appearing spindle cells and scattered inflammatory cells—mainly lymphocytes and plasma cells (Fig. 2a, b). On immunohistochemical staining, the tumor was positive for vimentin and anaplastic lymphoma receptor tyrosine kinase (ALK) and showed a positive signal pattern on ALK fluorescence in situ hybridization (FISH) test (Fig. 2c, d). Finally, IMT was diagnosed based on these findings. The resection margins and cytological examination of the pericardial fluid were negative for malignant cells. The patient did not undergo additional postoperative treatment, and no sign of recurrence was observed at the 6-months postoperatively. Regular follow-up imaging examinations have been planned for the patient.

## Discussion

To date, IMT has been ambiguously known as an inflammatory pseudotumor, fibromyxoid tumor, plasma cell granuloma, inflammatory fibrosarcoma, or pseudosarcomatous myofibroblastic tumor. However, Gleason et al. suggested that IMT should be clearly distinguished from various neoplastic and reactive lesions and that the unqualified designation of “inflammatory pseudotumor” should be avoided [2].

IMT mostly develops in children and young adults, with the main site of onset being the lungs and abdominopelvic cavity. Several studies have reported the onset of IMT in various somatic soft tissues. As described in this report, an IMT originating from the aorta is extremely rare, although less than 50 cardiac IMTs have been reported thus far [3].

The clinical symptoms of an IMT can vary depending on the infiltrated organ and mass effect. In 15–30% of the patients, general inflammatory symptoms, including fever, weight loss, and malaise, are commonly observed; however, asymptomatic development of the mass is also a distinct possibility [1, 4]. Cantera et al. reported that IMTs are often suspected to be malignant neoplasms, based on radiologic features, while they are not in reality. Their predominant radiologic presentation is as solid, irregular, and well-defined masses, although these findings are non-specific [5].

A definitive diagnosis of IMT is made by histologic examination. The characteristic feature of IMT is proliferation of myofibroblastic spindle cells in a myxoid to collagenous stroma with prominent inflammatory infiltrates, including plasma cells and lymphocytes and/or eosinophils. Immunohistochemical analysis can be helpful, and the tumor is often positive for smooth muscle actin, vimentin, and desmin. Approximately 50% of the IMTs exhibit positivity for ALK, and this is reported to affect the prognosis; however, there is no consensus on this association, and ALK positivity is not always necessary for diagnosing IMTs [6].

While there are various treatment options, the gold-standard treatment for IMT has not been clearly established. The majority of the previous studies have recommended complete surgical resection, if possible [7]. While local recurrence and distant metastasis rates of extrapulmonary IMTs vary based on their location and resectability, the overall rates are reported to be 25% and 5%, respectively. Although recurrence is often observed within a year from the initial surgery, recurrence after 9 years was observed in a patient, suggesting the need for long-term follow-up [8].

## Conclusion

In this case report, we described a rare case of IMT originating from the ascending aorta in a female patient, which was successfully treated through surgery. Although this tumor originated from a vital organ, we expect a good prognosis after successful complete resection. Long-term follow-up is required for this patient.
